# Is There Any Correlation between Baseline Serum Cortisol Levels and Disease Severity in PCR-Positive COVID-19 Patients with and without Diabetes Mellitus?

**DOI:** 10.3390/vaccines10081361

**Published:** 2022-08-20

**Authors:** Müge Keskin, Sefika Burcak Polat, İhsan Ates, Seval İzdes, Hatice Rahmet Güner, Oya Topaloglu, Reyhan Ersoy, Bekir Cakır

**Affiliations:** 1 Endocrinology and Metabolism Department, Ankara City Hospital, 06800 Ankara, Turkey; 2 Endocrinology and Metabolism Department, Faculty of Medicine, Ankara Yıldırım Beyazit University, 06800 Ankara, Turkey; 3 İnternal Medicine Department, Ankara City Hospital, 06800 Ankara, Turkey; 4 Anesthesiology and Reanimation Department, Faculty of Medicine, Ankara Yildirim Beyazit University, 06800 Ankara, Turkey; 5 Infectious Disease and Clinical Microbiology Department, Faculty of Medicine, Ankara Yıldırım Beyazit University, 06800 Ankara, Turkey

**Keywords:** COVID-19, type 2 diabetes mellitus, serum basal cortisol, disease severity

## Abstract

Background: COVID-19 has caused a pandemic and is associated with significant mortality. The pathophysiology of COVID-19, affecting many organs and systems, is still being investigated. The hypothalamus, pituitary gland, and possibly adrenal glands are the targets of SARS-CoV-2 because of its angiotensin-converting enzyme 2 (ACE2) and transmembrane serine protease 2 (TMPRSS2) receptors expression. Hypocortisolemia can be seen in the postinfection period. COVID-19 infection tends to be severe in diabetic patients due to immune dysfunction. In this study, our aim was to investigate the relationship between basal cortisol levels and the course of COVID-19 infection in diabetic and non-diabetic patients. Methods: Our retrospective study included 311 PCR-positive COVID-19 patients over the age of 18 who were hospitalized in Ankara City Hospital Infectious Diseases Department or Intensive Care Unit (ICU) between 15 March 2020 and 15 May 2020. Serum basal cortisol, fasting plasma glucose (FPG), HbA1c values, and diabetes history were recorded within the first 24 h of hospitalization. The presence of pulmonary involvement was noted from the patients’ imaging records. Pregnant and breastfeeding women, patients with chronic liver disease or chronic kidney disease, and patients who were already using steroids or had started COVID-19 infection treatment within the 72 h before blood collection were excluded from the study. Results: Of the 311 patients, 100 had Type 2 Diabetes Mellitus (T2D), while 211 did not. The age, serum basal cortisol, and glucose levels of the patients with T2D (64.51 ± 12.29, 19.5 ± 13.12, and 143.5 (77–345)) were higher than those of the patients without T2D (46.67 ± 16.38, 15.26 ± 8.75, and 96 (65–202)), and the differences were statistically significant (*p* = 0.004, *p* = 0.004, and *p* < 0.001, respectively). The basal cortisol values of the ICU patients (27.89 (13.91–75)) were significantly higher than those of the ward patients (13.68 (1.48–51.93)) and patients who were transferred to the ICU from the ward due to worsening conditions (19.28 (7.74–55.21)) (*p* < 0.001 and *p* = 0.007, respectively). The factors affecting ICU admission were determined to be age, T2D history, basal cortisol, and elevation in FPG using univariate logistic regression analysis. In the multiple logistic regression analysis, age, basal cortisol level, and infiltrative involvement in thorax CT were determined to be the risk factors affecting intensive care admission. Conclusion: High basal cortisol levels in patients with T2D may predict the severity of COVID-19 infection or mortality. Although high basal cortisol levels are among the risk factors affecting ICU admission, patients with COVID-19 should also be evaluated in terms of clinical and laboratory findings and relative adrenal insufficiency.

## 1. Introduction

COVID-19 infection, caused by severe acute respiratory syndrome coronavirus-2 (SARS-CoV-2) and reported with the first case in Wuhan in December 2019, has become an alarming and important health problem all over the world and has caused a pandemic with a high rate of contagion and over 557 million cases [1]. The clinical course of COVID-19 disease varies in severity depending on the individual’s genetic susceptibility and comorbidities [2]. Although the mortality rate differs according to country, it may reach 20% [3]. Studies have shown that the presence of diabetes mellitus and individual degree of hyperglycemia are associated with the severity of COVID-19 infection and increased mortality in patients with COVID-19 [4]. Hyperglycemia may promote viral proliferation. In individuals with diabetes mellitus, the disruption of glycemic regulation is a typical complication of COVID-19 infection. In addition, they have decreased natural killer cell (NK) activity and impaired immune functions [5]. There is not much proven information on the hypothalamic–pituitary effect of COVID-19. In a report from France, it was reported in autopsy studies that the hypothalamus is the target of COVID-19 by showing the excessive expression of angiotensin-converting enzyme 2 (ACE2) and transmembrane serine protease 2 (TMPRSS2) in the hypothalamus, especially in the paraventricular nucleus [6]. It has been shown that secondary adrenal insufficiency develops by SARS-CoV-2, the fact that SARS-COV-2 has amino acid sequences like the adrenocorticotropic hormone (ACTH), and the immune response of the host cross-reaction with ACTH. It was thought that this might result in a decrease in cortisol response due to ACTH [7]. In a postmortem case series of patients with COVID-19, a significant adrenal injury was demonstrated, and the importance of adrenal glands in immune regulation and the necessity of monitoring for adrenal insufficiency in acute infection and during the recovery period was emphasized [8]. Our aim in the present study was to evaluate serum basal cortisol values in patients hospitalized with the diagnosis of COVID-19 infection in the ward and intensive care unit (ICU) and to investigate whether it differs between patients with and without diabetes and the importance of disease severity.

## 2. Materials and Methods

### 2.1. Study Design and Participants

Our retrospective study included 311 patients over the age of 18 with PCR-positive COVID-19 who were hospitalized in Ankara City Hospital Infectious Diseases Department and ICU between 15 March 2020 and 15 May 2020. Serum basal cortisol, fasting blood sugar, and HbA1c values within the first 24 h of hospitalization and history of Type 2 Diabetes Mellitus (T2D) were recorded. The presence of pulmonary involvement was noted from the patients’ imaging records. Blood samples for cortisol measurements were taken between 08:00 and 09:00 A.M after an overnight fast. Serum cortisol levels were analyzed by chemiluminescence immunoassay with a Simens cortisol kit (Simens Atellica Hormone Analyzer, Tarrytown, NY, USA). Blood samples taken for ACTH measurement need to be carried by cold chain, so they could not be studied because there was no equipment to do this in the infection department. Pregnant and breastfeeding women, patients with chronic liver disease or chronic kidney disease, and patients who had started steroid treatment 72 h before blood sampling were excluded from the study. The patients were divided into two groups: those with and without T2D. According to the severity of the disease, ward patients were grouped as intensive care patients and patients whose condition worsened and were transferred from the ward to the ICU. According to the WHO, the severe patient group that has pneumonia plus one of the following, respiratory rate > 30 breaths/min or severe respiratory distress or SpO2 < 90% in room air, has acute respiratory distress syndrome. Patients without evidence of pneumonia or hypoxia were included in the mild group, while patients with signs and symptoms of pneumonia but no signs of severe pneumonia were considered moderate [9]. According to the severity of the disease, the group of patients who were transferred and hospitalized in the ICU was grouped as those with severe and critical disease, while patients hospitalized in the ward were grouped as those with mild and moderate disease. 

### 2.2. Ethical Approval:

The study was conducted in accordance with the principles of the Declaration of Helsinki. Ethics committee approval was obtained from Ankara City Hospital (Ethics Committee Approval No: E1-22-2701).

### 2.3. Statistical Analysis 

Continuous variables were indicated as mean ± standard deviation and/or median (min-max), and categorical data were indicated as numbers and percentages. Normality analyses of the continuous variables were performed using the Kolmogorov–Smirnov goodness of fit test. In the analyses between the two groups that matched the normal distribution, the *t*-test was used in independent groups, one-way analysis of variance (post hoc: Bonferroni) in analyses between three groups, the Mann–Whitney U test in analyses of unsuitable variables between two groups, and the Kruskal–Wallis test (post hoc: Mann–Whitney U test with Bonferroni correction) in analyses between three groups. The comparison of categorical data was carried out with the chi-square test and Fisher’s exact test.

Independent predictors of risk prediction using possible factors identified in previous analyses were first examined using univariate logistic regression analysis (enter method). For the multivariable model, the variables of age, sex, and basal cortisol and FPG values that were significant in univariate analyses and/or clinically significant; the presence of T2D; and the presence of infiltration in thorax CT were analyzed by multiple logistic regression analysis (backward LR). The Hosmer and Lemeshow test, omnibus tests of model coefficient, and Nagelkerke R square values were given for model fit and significance. The analyses were performed with IBM SPSS version 26.0 (IBM Corporation, Armonk, NY, USA) and the statistical significance level was set at *p* < 0.05.

## 3. Results

The mean age of the 311 patients was 52.41 ± 17.31 years, 47.9% of them were female, 52.1% were male, and 32.2% had T2D. While 90.4% (n = 281) of the patients were ward patients, 4.2% (n = 13) were ICU patients and 5.5% (n = 17) were transferred from the ward to the ICU during the follow-up due to the worsening of their general condition. While 97.4% of the patients were discharged after recovery, the COVID-19 infection resulted in mortality in 2.6% of patients. The mean HbA1c value measured in 83% of the patients with T2D was 7.91 ± 1.64. The mean baseline cortisol value of all patients was 16.62 ± 10.53 µg/dL and the mean FPG value was 118.97 ± 49.12 mg/dL (Table 1).

Some 82% of those with T2D were hospitalized in the ward, 9% in the ICU, and 9% of them were transferred to the ICU from the ward; for non-diabetics, those rates were 94.3%, 1.9%, and 3.8%, respectively, and the difference was statistically significant (*p* = 0.002). Age and basal cortisol and FPG levels [64.51 ± 12,29, 19.5 ± 13.12, and 143.5 (77–345)] were higher in the patients with T2D than in the patients without T2D [46.67 ± 16.38, 15.26 ± 8.75, and 96 (65–202)], and the differences were statistically significant (*p* = 0.004, *p* = 0.004, and *p* < 0.001, respectively) (Table 2).

According to the univariate logistic regression analysis performed to determine the risk factors affecting ICU admission, a 1-unit increase in age significantly increased the risk of admission to the ICU 1.09-fold (OR = 1.089, 95% GA = 1.054–1.125, *p* < 0.001), the presence of T2D significantly increased the risk of ICU admission 3.6-fold (OR = 3.640, 95% GA = 1.678–7.897, *p* = 0.001), a 1-unit increase in basal cortisol values significantly increased the risk of ICU admission 1.07-fold (OR = 1.076, 95% GA = 1.041–1.112, *p* < 0.001), and a 1-unit increase in glucose values increased the risk of ICU admission by 1.008-fold (OR = 1.008, 95% GA = 1.002–1.014, *p* = 0.008).

In the multivariate logistic regression model, the variables of age, basal cortisol and presence of infiltration remained significant and were found to be associated with poor prognosis. Accordingly, it was determined that a 1-unit increase in age significantly increased the risk of ICU admission 1.09-fold (OR = 1.091, 95% GA = 1.051–1.132, *p* < 0.001), a 1-unit increase in basal cortisol values significantly increased the risk of ICU admission 1.06-fold (OR = 1.063, 95% GA = 1.024–1.102, *p* = 0.001), and the presence of infiltration disease on thorax CT significantly increased the risk of ICU admission 3.9-fold (OR = 3.998, 95% GA = 1.198–13.346, *p* = 0.024) (Table 3). 

## 4. Discussion

In our study, serum basal cortisol levels were significantly higher in the patients with T2D than in the non-diabetic patients in the acute period of COVID-19 infection [respectively, 19.5 ± 13.12 vs. 15.26 ± 8.75]. We think that the high cortisol level of patients with T2D may be associated with disease severity and mortality risk, compatible with the literature. Patients with T2D are at high risk of severe COVID-19 infection [10]. Interleukin-6 (IL-6) is the key cytokine associated with COVID-19 disease severity, and high IL-6 levels have been detected in hyperglycemic patients with COVID-19 [11,12]. Moreover, in a meta-analysis of 15 prospective studies, high IL-6 levels were associated with an increased risk of T2D [13]. IL-6 has been shown to be a potent activator of the hypothalamic–pituitary–adrenal (HPA) axis in humans [14]. In the first population-based study examining the relationship between basal cortisol activity and inflammatory cytokines (IL-6, TNF-alpha, and IL-10), high IL-6 level was associated with higher daily cortisol level, and this relationship was found only with IL-6 [15]. 

In the present study, the factors affecting ICU admission were age, T2D history, basal cortisol levels, increased glucose levels and infiltrative involvement in thorax CT according to the univariate logistic regression analysis. In the multiple logistic regression analysis created by selecting these variables, the risk factors affecting ICU admission were age, high basal cortisol levels, and infiltrative involvement in thorax CT. There are studies in the literature showing serum cortisol as a predictor of COVID-19 disease severity. In a meta-analysis of 7 valid studies, it was shown that increased cortisol level is associated with increased mortality in community-acquired pneumonia, and it was concluded that it might be a prognostic marker in severe cases [16]. Pal R et al. advocated that serum cortisol concentrations can predict mortality risk, but due to individual differences in cortisol dynamics, cortisol levels should be evaluated by multivariate and survival analysis by grouping according to disease severity [17]. Tan et al. supported these findings in their study. They found for the first time that a significant acute basal cortisol stress response occurred in patients with COVID-19, and high cortisol concentrations were associated with increased mortality. They concluded that cortisol is a better predictor of COVID-19-related laboratory markers such as CRP, D-dimer, and neutrophil-to-lymphocyte ratio (NLR) [18]. Dashatan et al., in their meta-analysis consisting of 5 observational studies and 6 case reports, found high cortisol levels in patients with severe COVID-19 [19]. In a recent study, morning salivary cortisol was similar between the control group and patients with COVID-19, and evening and nighttime cortisol levels were high and correlated with IL-6 levels [20]. That might be explained by the distorted diurnal rhythm of cortisol in acute stress resulting in high midnight serum and salivary cortisol levels. 

Prolonged HPA activation in critical illness may result in hypercortisolemia or hypocortisolemia [21]. Although a definite threshold basal cortisol value is not recommended for the diagnosis of adrenal insufficiency associated with critical illness, it is possible to exclude the diagnosis of adrenal insufficiency if serum cortisol is >34 µg/dL [22]. In our study, the mean basal cortisol level of critically ill COVID-19 patients and intensive care patients was 27.9 (13.9–75) µg/dL, while the basal cortisol level of the patients who were transferred from the ward to the ICU due to worsening conditions was 19.2 (7.7–55.2) µg/dL. In this case, relative adrenal insufficiency may also occur in these patients. Diagnosis of adrenal insufficiency in critically ill patients is difficult, and clinically, basal cortisol level and corticotropin stimulation tests may be required in some patients [23].

In the initial phase of critical illness, physiological doses of glucocorticoids support the immune system, after which corticosteroid insufficiency may occur due to critical illness by blunting of the HPA axis [24]. In recent studies, the reason for the high cortisol at the onset of critical illness has been shown to be decreased ACTH levels, related to other mechanisms such as low cortisol-binding protein (CBG), disruption in the diurnal rhythm of cortisol and ACTH, and tissue-specific glucocorticoid resistance, which is now called critical illness-associated corticosteroid insufficiency (CIRCI) [25]. Since our study is a retrospective study, basal cortisol levels were examined, and basal values are a guide for other studies. Gibbison et al. evaluated the HPA axis after cardiac surgery and found a decrease in ACTH and an increase in non-pulsatile cortisol secretion in critically ill patients and concluded that factors other than the HPA axis might affect cortisol secretion in these patients [26]. The RECOVERY study provided evidence that 6-mg dexamethasone treatment for the first ten days reduces 28-day mortality in patients with COVID-19 requiring respiratory support [27]. Moreover, data from patients recovering from SARS suggest that hypocortisolism may develop gradually as a late complication several weeks after infection [28]. It may also develop as a late complication of hypocortisolism in SARS-CoV-2, but it has not been proven yet. 

Limitations of this study: Since the study was retrospective and the necessary equipment for ACTH transport could not be provided in the infection ward, ACTH levels could not be measured. Corticotropin stimulation could not be tested in terms of relative adrenal insufficiency.

## 5. Conclusions

Our study is the first in the literature to evaluate the relationship between the cortisol level and severity of infection and mortality in the acute phase of COVID-19 infection in diabetic patients. Although the basal cortisol level of diabetic patients was higher than that in non-diabetic patients, the diagnosis of adrenal insufficiency should be kept in mind during the acute, healing, and postinfectious periods of COVID-19 infection.

Further studies are needed on this subject due to the fact that diabetic patients are seen more commonly in risk groups in terms of COVID-19 infection and when seen due to the risk of serious illness or even fatal progression. More detailed studies are needed about the HPA axis in diabetic patients with COVID-19 and detailed molecular studies evaluating the ACTH, cortisol pulsatile secretion, and cortisol-IL-6 relationship. Studies on whether cortisol can be an inflammatory marker in acute infection will also shed light on the points that have not yet been clarified in the pathophysiology of COVID-19.

## Figures and Tables

**Table 1:** Demographic and clinical characteristics of the patients.

		N	%
**Sex**	**Female**	149	47.9
	**Male**	162	52.1
**T2D status**	**Non-T2D**	211	67.8
	**T2D**	100	32.2
**Hospitalization status**	**Ward**	281	90.4
	**ICU**	13	4.2
	**Deteriorating patients**	17	5.5
**Outcome**	**Death**	8	2.6
	**Recovering patients**	303	97.4
**Infiltration on thorax CT**	**No infiltration**	90	28.9
	**Confirmed infiltration**	215	69.1
	**No CT**	6	1.9
**Total**		**311**	**100**

**Table 2:** Comparison of age, sex, basal cortisol, FPG, hospitalization status, and survey and tomography findings of patients with and without diabetes.

		T2D Status				*p*
		Non-T2D (n = 211)		T2D (n = 100)		
		n	%	n	%	
**Age (mean ± SD), years**		46.67 ± 16.8	**-**	64.51 ± 12.9	**-**	0.004 *
**Baseline cortisol (mean ± SD), µg/dL**		15.26 ± 8.75	**-**	19.5 ± 13.12	**-**	0.002 *
**FPG [median (min-max)], mg/dL**		96 (65–202)	-	143.5 (77–345)	-	<0.001 **
**Sex**	**Female**	99	46.9	50	50.0	0.612 ***
	**Male**	112	53.1	50	50.0	
**Hospitalization status**	**Ward**	199	94.3	82	82.0	0.002 ***
	**ICU**	4	1.9	9	9.0	
	**Deteriorating patients**	8	3.8	9	9.0	
**Outcome**	**Death**	2	0.9	6	6.0	0.015 ****
	**Recovering ** **patients**	209	99.1	94	94.0	
**Infiltration on thorax CT**	**No infiltration**	68	32.2	22	22.0	0.031 ***
	**Confirmed infiltration**	137	64.9	78	78.0	
	**No CT**	6	2.8	0	0	
**Total**		211	100.0	100	100.0	

* Independent samples *t*-test; ** Mann–Whitney U test; *** Chi-square test; **** Fisher’s exact test.

**Table 3:** Multiple logistic regression analysis to determine risk factors affecting ICU admission in COVID-19 patients.

	B	SE	Exp(B)	95% GA	*p*
**Age, years**	0.087	0.019	**1.091**	**1.051–1.132**	**<0.001**
**Basal cortisol**	0.061	0.019	**1.063**	**1.024–1.102**	**0.001**
**Infiltration on thorax CT (ref: na)**	1.386	0.615	**3.998**	**1.198–13.346**	**0.024**
**Constant**	−9.921	1.568	0.000		**<0.001**

* Multiple logistic regression analysis (backward LR); omnibus tests of model coefficient < 0.001; Nagelkerke R square = 0.355; Hosmer and Lemeshow test = 0.105.

## Data Availability

The data supporting this study’s findings are available on request from the corresponding author. The data are not publicly available due to privacy or ethical restrictions.
